# Human norovirus disturbs intestinal motility and transit time through its capsid proteins

**DOI:** 10.1371/journal.ppat.1012710

**Published:** 2024-11-27

**Authors:** Arno Cuvry, Lorane Molineaux, Roberto Gozalbo-Rovira, Johan Neyts, Peter de Witte, Jesús Rodríguez-Díaz, Joana Rocha-Pereira

**Affiliations:** 1 KU Leuven, Department of Microbiology, Immunology and Transplantation, Rega Institute, Virus-Host Interactions & Therapeutic Approaches (VITA) Research Group, Leuven, Belgium; 2 KU Leuven, Translationeel Onderzoek van Gastro-enterologische Aandoeningen (TARGID), Leuven, Belgium; 3 Department of Microbiology, School of Medicine, University of Valencia, Valencia, Spain; 4 KU Leuven, Department of Microbiology, Immunology and Transplantation, Rega Institute, Virology, Antiviral Drug & Vaccine Research Group, Leuven, Belgium; 5 KU Leuven, Laboratory for Molecular Biodiscovery, KU Leuven-Department of Pharmaceutical and Pharmacological Sciences, Leuven, Belgium; Heidelberg University, GERMANY

## Abstract

Human norovirus (HuNoV) accounts for over 700 million cases of gastroenteritis annually. Episodes of HuNoV disease are characterized by vomiting and diarrhea as the two most prominent symptoms. Despite its prevalence, our understanding of the pathophysiological mechanisms triggered upon HuNoV infection is limited, mainly due to a lack of suitable animal models. Our aim was to use the recent HuNoV zebrafish larvae model to study the effect of HuNoV infection on intestinal motility and investigate whether one viral protein could act as an enterotoxin, as seen with rotavirus. We studied whether HuNoV infection affects the contraction frequency of the intestinal bulb and the posterior intestine as well as the transit time. Infection of larvae, following injection of a HuNoV GII.4-containing stool sample in the yolk, resulted in an increased contraction frequency in the intestinal bulb. A comparable effect was observed in serotonin-treated larvae, corresponding to the natural function of serotonin. The higher replication efficacy of HuNoV GII.4 likely explains why they have a more marked effect on gut motility, when compared to other genotypes. Additionally, transit time of fluorescent food was prolonged in HuNoV GII.4 infected larvae, suggesting a loss of coordination in bowel movements upon infection. To identify the proteins responsible for the effect, individual HuNoV non-structural proteins and virus-like particles (VLPs) were injected intraperitoneally (ip). VLPs carrying VP1/VP2, but not those with only VP1, induced increased contraction frequencies in the intestinal bulb in a dose-dependent manner. In conclusion, our findings suggest that the viral capsid and potentially the minor capsid protein VP2 play a crucial role in the aetiology of symptoms associated with HuNoV, potentially acting as a viral enterotoxin. This work contributes to the understanding of the pathophysiological mechanisms in HuNoV-induced disease and further attests zebrafish as a valuable HuNoV disease model.

## Author summary

Human norovirus causes more than 700 million cases of gastroenteritis every year. Episodes of HuNoV disease are characterized by vomiting and diarrhea as the two most prominent symptoms. Despite its global prevalence, our understanding of the underlying mechanisms causing human norovirus disease are limited due to a historical lack of proper animal models. Recently, we developed a new small animal model for human norovirus in zebrafish larvae. Here, we used the optical transparency during the early larval stages of zebrafish larvae to study the effect of human norovirus infection on the intestinal motility and transit time, and to potentially identify a new viral enterotoxin, as has been done for human rotavirus. We found that the contraction frequency of a specific part of the zebrafish intestine, the intestinal bulb, increased upon human norovirus infection. Additionally, human norovirus infection caused a prolonged transit time after feeding, indicating a loss of coordination in bowel movements upon infection. Finally, we show that it are the viral capsid proteins that play a crucial role in inducing these effects. Overall, our study provides new insights into the disease mechanism of human norovirus.

## Introduction

Human norovirus (HuNoV) infections are the most common cause of acute gastroenteritis among all age groups, with nearly 700 million infections worldwide every year [1]. In healthy adults, a typical HuNoV infection is self-limiting with the main symptoms being vomiting and non-bloody diarrhea, while fever, abdominal cramps, nausea and myalgia are also common. Vulnerable populations like young children, the elderly and immunocompromised individuals are at a higher risk of suffering from dehydration, malnutrition and other complications and may succumb to disease [2,3]. Yearly, around 200 000 deaths are attributed to HuNoV infection, mainly children in developing countries. Immunocompromised patients often develop chronic HuNoV infection where diarrhea, vomiting and viral shedding can persist for months to years [4].

The underlying disease mechanism of how a HuNoV infection results in diarrhea and vomiting is still poorly understood. For murine norovirus (MNV), diarrheagenic strains tend to replicate in the intestinal submucosa and spread to extraintestinal tissues such as the liver and spleen, which is suggested to correlate with its dual cell tropism (immune cells and intestinal cells) [5]. Moreover, capacity of the VP1 protein, the major capsid protein responsible for receptor-binding, to undergo environmentally-triggered contraction of its P domain onto the particle shell has been reported to be a key determinant of the viral diarrheagenic potential [6]. This suggest that the norovirus capsid proteins and their effect on the viral binding capabilities partly determine the diarrheagenic potential of each norovirus strain.

While at least eight different bacterial enterotoxins associated with foodborne illness have been described, only two gastroenteritis-related viral enterotoxins are currently known, i.e. the human rotavirus (HRV) non-structural protein 4 (NSP4), and the turkey astrovirus capsid protein. More specifically, after infection of intestinal enterocytes by HRV, the main cause of acute gastroenteritis in young children, NSP4 is initially formed and through its viroporin function triggers Ca^2+^ release from the ER into the cytoplasm resulting in a Cl^-^ efflux, subsequent water loss and eventually electrolyte disturbance (e.g. hypokalemia, hypernatremia) [7,8]. The NSP4 released from the infected cells initiates a phospholipase C pathway in neighbouring enterocytes through paracrine action, eventually resulting as well in Ca^2+^-release. Besides enterocytes, HRV and paracrine NSP4 also affect enterochromaffin cells, the most common intestinal enteroendocrine cells [9]. Here, increased Ca^2+^-concentration by NSP4, like cholera toxin (CT), stimulates the release of serotonin at basolateral side of the epithelium, which trigger the intrinsic and extrinsic afferent nerves of the ENS. In turn, the ENS induces increased fluid secretion, disturbs the intestinal motility and triggers the central nervous system, leading to the sensation of nausea and activation of the vomiting centre [9,10].

Additionally, oral administration of purified recombinant turkey astrovirus capsid protein in turkey poults let to a dose- and time-dependent induction of acute diarrhea [11]. This acute diarrhea was a result of disruption of the intestinal barrier function, as was previously also seen to be the effect of human astrovirus in vitro [12].

We hypothesize that HuNoV, like HRV and astrovirus, encodes an enterotoxin which is one of the main responsibles for the clinical symptoms associated with HuNoV infection, through dysregulation of the intestinal physiology. This hypothesis is based in the similitude to HRV disease symptoms, and the lack of evidence of the presence of other disease inducing mechanisms such as massive lysis of enterocytes or a high-level of inflammation that would disrupt intestinal permeability and homeostasis. To study the effect of HuNoV and its viral proteins on the intestinal physiology, the zebrafish larva model established by our team has been used [13].

Zebrafish (*Danio Rerio*) are a small vertebrate model that we and others have brought forward as a virus infection model. Its key advantages are their optical transparency during early larval stages and their fast biological development. We previously used zebrafish larvae to establish the first small robust *in vivo* model for HuNoV. Following the intra-yolk injection of a stool suspension obtained from a Human Norovirus (HuNoV) infected patient, zebrafish larvae exhibit sustained viral replication for a period of up to six days. This provides an opportunity for in-depth investigation into various aspects of HuNoV biology [13].

The intestinal architecture of zebrafish strongly resembles that of humans, with irregular folds resembling the mammalian villi, and consists of absorptive enterocytes, goblet cells and enteroendocrine cells, while Paneth cells are lacking [14]. The zebrafish intestine is divided into three morphologically and functionally distinct segments: the anterior intestinal bulb (IB), the mid-intestine (MI) and the posterior intestine (PI) [15]. The enlarged IB exhibits high expression levels of digestive enzymes, aligning with its principal roles in food degradation and nutrient absorption, akin to the functionalities observed in the human stomach and small intestine [15]. Further along the intestine, the MI and the anterior part of the PI share similarities to the mammalian small intestine and are responsible for lipid and protein absorption. The most posterior segment of PI shares greater resemblance with the mammalian colon and facilitates ion and water reabsorption processes. [15–17]. Furthermore, the zebrafish intestine is surrounded by a smooth muscle layer consisting of circular and longitudinal smooth muscles connected directly to the mucosal layer without an intermediate submucosal layer or an analogue to the mammalian muscularis mucosae [15].

Larvae of 3 days post fertilization (dpf) present a continuous lumen with sporadic, spontaneous, non-propagating movements. However not all intestinal cell types are yet differentiated and invaginations have not yet formed. By 5 dpf, the intestine is fully functional with a regular intestinal motility pattern and all cell types are present [18]. This timepoint normally marks the onset of exogenous feeding in nature.

The overall goal of this work was to determine whether, like HRV and astrovirus, HuNoV encodes for an enterotoxin within its structural or non-structural proteins. To that end, we first investigated whether a HuNoV infection induces changes in the intestinal motility and transit time of 5-dpf zebrafish larvae, and secondly whether an increase in motility is directly induced by one or more of the viral capsid or the viral non-structural proteins.

## Results

### Human norovirus infection increases intestinal contraction frequency

**Fig 1:**
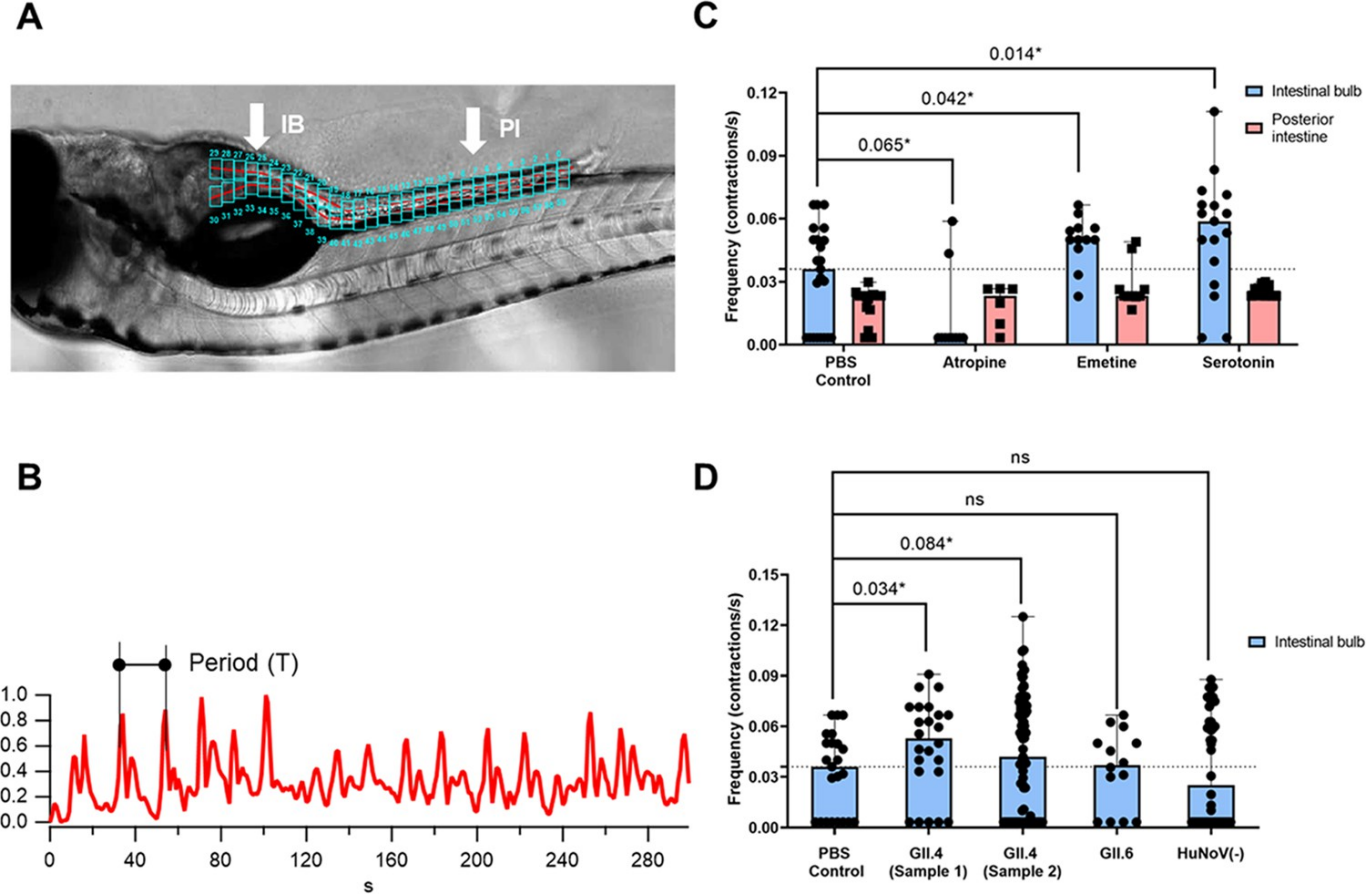
HuNoV infection increases intestinal contraction frequency in 5 dpf larvae. **A)** An agar-embedded 5-dpf zebrafish analysed in Igor Pro with the area of analysis (AOA) defined by the two red lines and the individual regions marked by the cyan boxes (0–59). Contraction frequency was analyzed in the intestinal bulb (IB) and posterior intestine (PI). **B)** Representative data output from an individual region of evaluation. Graphs are plotted based on the change in pixel intensity (y-axis) over time (x-axis). For data analysis, we took the frequency [1/period (T)] **C)** The effect of atropine (20 μM, n = 11;6), emetine (500 μM, n = 12;10), serotonin (200 μM, n = 17;12) on intestinal contraction frequency compared to the PBS control group (n = 23;15) in the intestinal bulb and posterior intestine. (n = n_IB_;n_PI_). **D)** The effect of GII.4 HuNoV infection–sample 1 (n = 24), and sample 2 (n = 81), GII.6 HuNoV infection (n = 14) and injection of a HuNoV-negative stool sample (n = 50) on the intestinal contraction frequency compared to the PBS control group (n = 23) in the intestinal bulb and posterior intestine. *p ≤ 0.1 P-values are shown compared to control. The dotted line represents the median value of the control group.

To assess the effect of HuNoV infection on intestinal motility, we established a method to quantify the intestinal contractions in zebrafish larvae with live imaging. To that end, 5-dpf larvae were anesthetised and embedded in agar in a chambered slide, allowing the correct positioning for acquisition of a 5 min video of 1 frame per second. We verified our method by quantifying the intestinal contraction frequencies after pharmacological treatment with compounds that either inhibit (atropine) or increase (serotonin, emetine) intestinal motility. The intestinal motility was analysed using two software tools: Danioscope (Noldus, The Netherlands) and Igor Pro (Wavemetrics, USA). Both tools use differences in mean grey value in between frames to calculate contraction peaks. For this evaluation, we selected two regions along the intestinal tract: the centre of the intestinal bulb (IB) and the posterior intestine (PI), between the 6^th^ and 7^th^ vertebra (**Figs 1A and S1**). Periodic intervals were calculated from the output data and further converted to frequency [contractions (c)/s]) for all data visualisations (**Fig 1B**). As expected, treatment with atropine reduced the median intestinal contraction frequency in the IB (0.003 c/s) compared to the control (0.036 c/s) (**Fig 1C**). Conversely, addition of serotonin and emetine to the swimming water resulted in an increase of the contraction frequency in the IB to 0.059 c/s and 0.050 c/s, respectively. Measurements in the PI showed no changes in any of the treatment conditions. For this reason, we decided to focus on the IB region in subsequent experiments. Overall, data analysis resulted in overlapping results from both software tools and showed that our method accurately measures the contraction frequency of the zebrafish larval intestine.

Next, it was assessed whether HuNoV infection had an impact on the intestinal motility of larvae. To that end, zebrafish larvae were infected at 3 dpf with two different HuNoV GII.4 stool samples, and their gut motility was assessed at the peak of viral replication (2 days post infection (dpi), and 5 dpf) (**Fig 1D**) [13]. For HuNoV GII.4 sample 1 and 2, we found a significant increase in median contraction frequency in infected larvae (0.053c/s and 0.047c/s, respectively), comparable to the values observed in serotonin- and emetine-treated larvae. Thus, while zebrafish larvae do not present obvious signs of disease upon HuNoV infection (e.g. edema, loss of posture, altered heart rate), they do display changes in intestinal motility upon HuNoV GII.4 infection, like what is described in the natural human host. However, after infection with HuNoV GII.6, no effect on the contraction frequency was observed. Of note, the replication efficiency on 2 dpi of GII.6 was lower by ~1 log_10_ when compared to GII.4, akin to what we have previously reported [13] (**S2 Fig**). This could explain why the effect of GII.6 on gut motility was not significantly different from the PBS-injected control fish.

Moreover, to exclude that other stool-related factors are driving these changes in intestinal motility, we measured intestinal contractions after injection with a negative stool sample, i.e. not containing HuNoV virus (HuNoV(-)). Here, no significant difference with the PBS control was observed (0.025 c/s).

### HuNoV infection prolongs intestinal transit time

**Fig 2:**
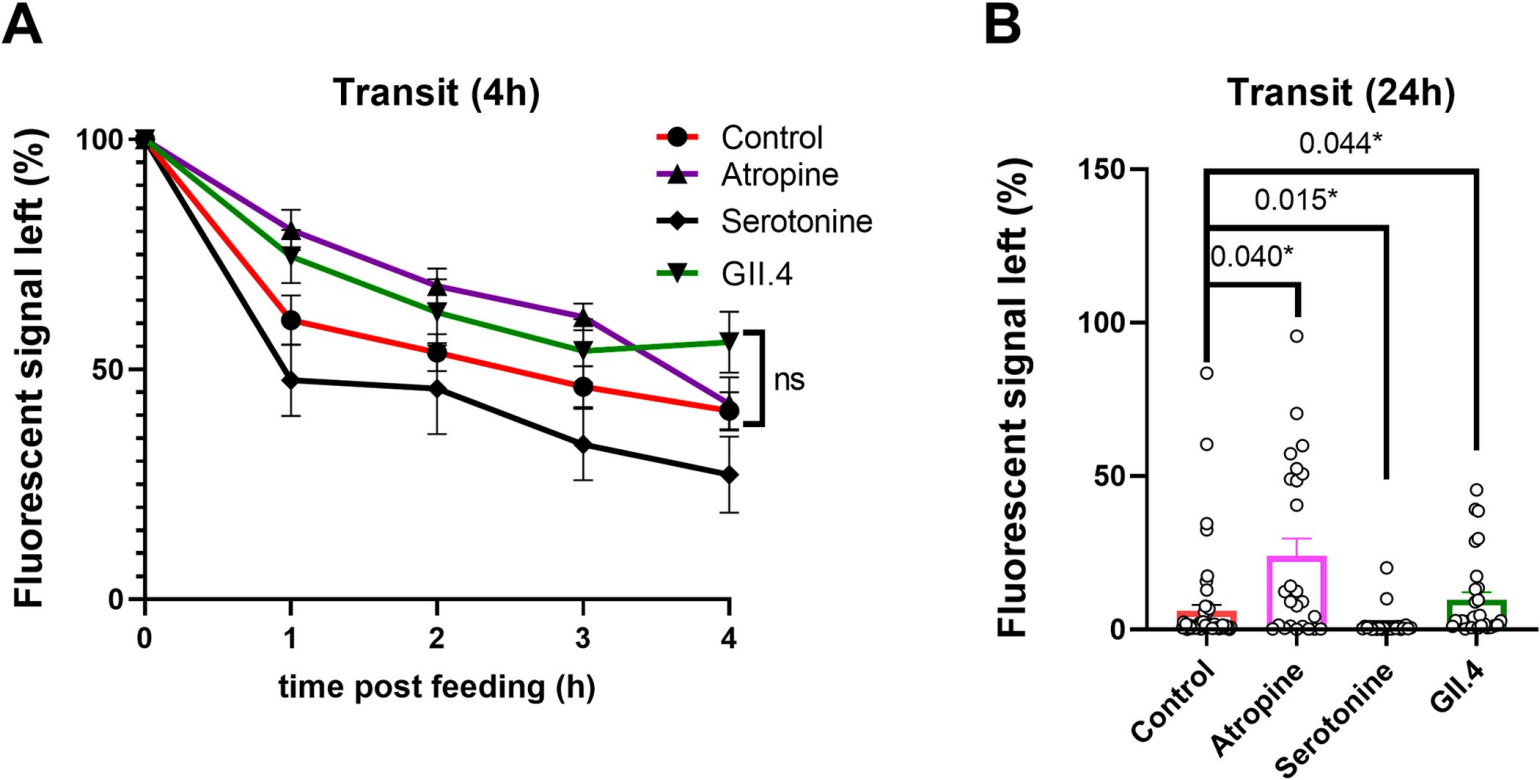
HuNoV infection slows down intestinal transit. **A)** Decrease of fluorescent signal over 4 hours in 5-dpf zebrafish larvae. Larvae were imaged every hour for 4 hours. Larvae were either non-treated (n = 14), continuously treated with atropine (20 μM, n = 6) or serotonin (200 μM, n = 8), or priorly infected with a GII.4 HuNoV at 3 dpf (n = 14). Two-way ANOVA test was performed. **B)** Remaining signal after 24 hours in controls (n = 55), serotonin- (n = 23) and atropine- (n = 25) treated and HuNoV-infected (n = 29) larvae. Mean ± SEM are presented. Kruskal-Wallis tests with multiple comparisons to the control group were performed. *p ≤ 0.1.

Next we studied whether HuNoV infections have an effect on the transit of food through the gastrointestinal tract of 5-dpf larvae. To that end, we developed an intestinal transit assay where the clearance of fluorescent food after feeding was quantified. Briefly, HuNoV-GII.4. infected larvae were fed a mixture of dry food and 1-μm fluorescent beads for 4 h before live imaging. While serotonin treatment accelerated the clearance of fluorescent food, treatment with atropine slowed it down (**Fig 2A**). Interestingly, HuNoV infection seemed to slow down the transit of food in infected larvae, when compared to non-infected controls. To have a more comprehensive view of this effect, we followed infected larvae individually for a period of 24 h (**Fig 2B**). Here, we obtained similar results, i.e. 62% more food remained in the intestine of HuNoV-infected larvae after 24 h compared to the control group, thereby confirming that HuNoV infection prolongs the transit time of zebrafish. Interestingly, we additionally observed a reduced number of HuNoV-infected larvae with a filled (fluorescent) IB after 4h feeding (**S3 Fig**).

### VP1/2 capsid proteins trigger increased intestinal contraction frequency

To identify whether one of the viral protein(s) could itself induce increased intestinal contractions upon a HuNoV infection, zebrafish larvae were inoculated with virus-like particles (VLPs) containing the VP1 and VP2 capsid proteins (or only VP1), or with the HuNoV non-structural (NS) proteins NS1/2 (N-term), NS4 (p22), NS6 (Pro) and NS7 (RdRp). NS3 (NTPase) and NS5 (Vpg) could not be expressed to high enough concentrations and were therefore not included. We confirmed the purity and the structure of the HuNoV VLPs and HuNoV non-structural proteins by transmission electron microscopy (**S4 Fig**) and Coomassie blue staining (**S5 Fig**).

**Fig 3:**
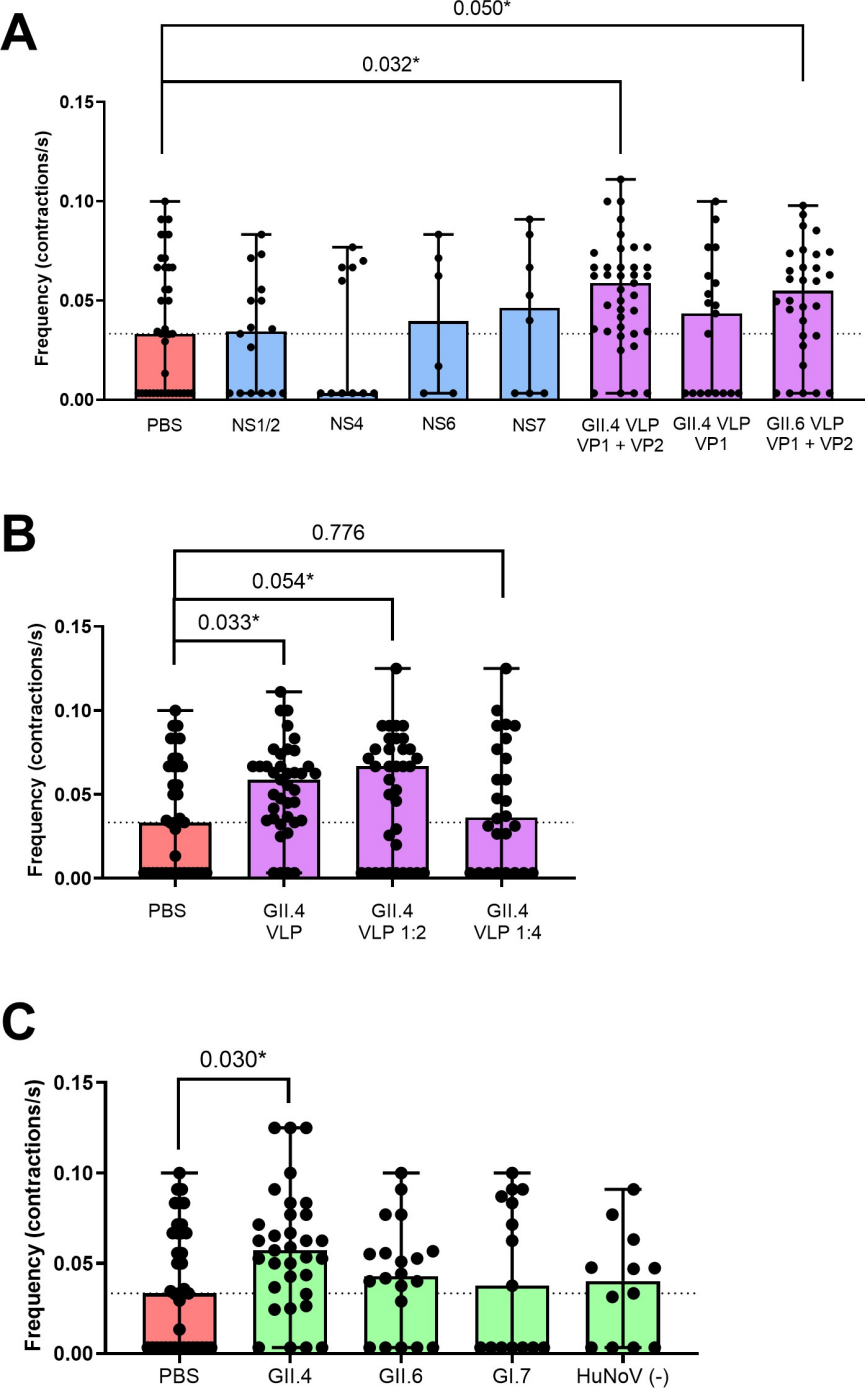
Intraperitoneal injection of VP1/VP2 VLPs increases intestinal contraction frequency. **A)** intraperitoneal injection of PBS (n = 38), viral non-structural (NS) proteins NS1/2 (n = 16), NS4 (n = 11), NS6 (n = 6), NS7 (n = 7), GII.4 VLPs consisting of VP1+VP2 (n = 38) or only VP1 (n = 19) and GII.6 VLPs consisting of VP1+VP2 (n = 30). **B)** intraperitoneal injection of GII.4 VLPs containing VP1/VP2 proteins, undiluted (n = 39), 1:2 (n = 36) or 1:4 (n = 25) diluted. **(C)** Intraperitoneal injection of HuNoV(+) stool containing GII.4 (n = 33), GII.6 (n = 14) GI.7 (n = 8) and HuNoV(-) stool (n = 12). *p ≤ 0.1. The dotted line represents the median value of the control group.

Here, the injection was performed intraperitoneally (ip) to ensure timely delivery of the proteins to the gastrointestinal tract before degradation, as opposed to yolk injection. The effect was monitored after 4 h, alike done by others with rotavirus NSP4 in mice pups [19]. Ip injection of the individual NS proteins did not affect intestinal motility nor did injection of GII.4 VLPs consisting of VP1. However, a clear increase in contraction frequency was observed after injection of GII.4 VLPs containing both VP1 and VP2 capsid proteins (0.059 c/s) (**Fig 3A**). Basal levels of PBS-injected controls was similar to what we report in Fig 1 (0.033 c/s). Next, we studied whether the observed effect was dose-dependent by injecting ip 1:2 and 1:4 dilutions of the GII.4 VP1/VP2 VLPs (**Fig 3B**). Whereas a 1:2 dilution induced a similar effect (0.067 c/s), no effect on the contraction frequency was detected with a 1:4 dilution (0.036 c/s). Surprisingly, ip-injection of GII.6 VP1/VP2 VLPs at the same concentration resulted in a similar increase in median contraction frequency (0.055 c/s) as seen with the GII.4 VP1/VP2 VLPs (**Fig 3A**).

Given that increased intestinal contractions were induced by the viral capsid, we next studied whether the same effect was achieved after ip injection of complete virus particles by using a HuNoV GII.4(+) sample 1 stool suspension. As expected, we observed an equal increase (0.057 c/s) in contraction frequency as with the GII.4 VP1/VP2 VLPs. There was little to no change in contraction frequency after ip injection of GII.6 or GI.7 virus samples (**Fig 3C**). This could be due to there being less virus particles present in these positive stool suspensions, when compared to the injected VLPs. As with the yolk injections, we excluded the effect of other stool-related factors on the contraction frequency by injecting a HuNoV(-) stool sample under the same conditions, which resulted in a contraction frequency alike the PBS control.

## Discussion

Understanding the cellular mechanisms and pathophysiology of HuNoV gastroenteritis has long been hindered by the lack of small animal models of HuNoV infection and disease. The zebrafish larvae model provides an ideal platform to study new aspects of HuNoV biology due to its unique characteristics. Here, we used the optical transparency of zebrafish larvae to study the effect of HuNoV infection on the intestinal motility by live imaging. Our work focussed on the quantifiable features of the gastrointestinal motility of zebrafish larvae since a direct visualization of diarrhea at the larval stage is not feasible, due to the small size of the excrements and their immediate dilution in the swimming water. In adult zebrafish, changes in faecal consistency caused by HRV infection could be distinguished [20]. Our current model utilizes yolk injection for virus inoculation; the yolk is present until ~5 dpf after which external feeding normally starts. A future alternative could be to establish a juvenile/adult zebrafish model in which we inoculate HuNoV by oral gavage [21].

Imaging processing software was used to measure intestinal contractions in zebrafish larvae. The natural contraction frequency we observed in the posterior intestine is in line with values previously reported [22–24]. Treatment with atropine drastically lowered the contraction frequency in the intestinal bulb, even to lower values than others have reported. However, our exposure time and concentration were higher, likely explaining the difference [24]. Treatment with serotonin and emetine increased contraction frequency in the same region. In summary, these controls validated our method as capable to clearly discriminate between higher and lower frequencies in the intestinal bulb of zebrafish larvae.

We detected that infection of the zebrafish larvae with HuNoV GII.4 resulted in an increase of contraction frequency in the intestinal bulb, similar to the values observed in serotonin-treated non-infected larvae. We started with HuNoV GII.4, since it is the most clinically relevant genotype, causing large outbreaks with symptomatic patients. However, we observed no such effect when using a HuNoV GII.6 genotype. This is probably explained by the superior replication efficacy of GII.4 viruses that reach ~10^7^ RNA copies/ larva at the peak of replication. Of note, we here studied in total two GII.4 strains/samples, and one GII.6 strain/ sample, while a more comprehensive testing of a large number of samples would be desirable to have a more complete view.

While HuNoV GII.4 infection resulted in increased contraction frequency in the intestinal bulb, we found that it prolonged transit time. In contrast, in serotonin-treated zebrafish larvae, increased contraction frequency correlated with a reduced transit time. This difference may be the result of disruption of the coordination of the surrounding smooth muscles. HuNoV was recently shown to infect enteroendocrine cells (EECs), one of the major serotonin-secreting cells in the intestinal epithelium [25]. Serotonin is one of the major neurotransmitters in the enteric nervous system, controlling smooth muscle contractions. Hence, HuNoV-triggered serotonin release after infection of EECs could contribute to this loss of smooth muscle coordination, akin what was described for HRV [9]. However, other neurotransmitters such as dopamine and relaxine are also known to (negatively) affect intestinal motility. It was recently shown in a 3D organoid model that response to this neurotransmitters is upregulated upon HuNoV infection [26]. The here observed prolonged transit time thus may be a result of the combined action of these neurotransmitters, impacted by HuNoV-infection.

Additionally, delayed gastric emptying (or gastroparesis), which has been observed as a consequence of HuNoV infection in the past [27], may also play a role in the observed prolonged transit time. Gastroparesis leads to bloating of the stomach and is believed to contribute to the high incidence of vomiting episodes in humans with viral gastroenteritis [28]. Likewise, MNV infection has been accompanied by gastric bloating in immunodeficient mice and fluid accumulation in the small intestine [29,30]. However, this could not be immediately linked to vomiting episodes, due to the absence of an emetic reflex in rodents, preventing an observable clinical outcome apart from diarrhea [31]. The slow transit in HuNoV-infected zebrafish larvae suggests delayed gastric emptying is indeed at play and that the induced contractions are of uncoordinated nature, without an anterograde propelling effect.

To understand whether one of the viral proteins of HuNoV can act as an enterotoxin, we injected HuNoV structural and non-structural proteins ip, to ensure timely delivery of the proteins in the gastrointestinal tract, thereby avoiding protein degradation. When expressed in cells, norovirus VP1 proteins form VLPs consisting of 180 VP1 monomers. When co-expressed with VP2 a VP1/VP2 VLP is formed consisting of 180 VP1 monomers interspersed with few VP2 proteins. Injection of GII.4 VLPs consisting of both VP1 and VP2 capsid proteins resulted in an increase of contraction frequency within four hours, a similar timeframe as described for rotavirus NSP4, known to be an enterotoxin [19]. However, the same effect was not observed when VLPs consisting of only VP1 were injected, suggesting that VP2 has a critical role in inducing this effect. We also confirmed the effect is dose-dependent, by injecting a serial dilution of VP1/VP2 VLPs.

While VLPs solely consisting of VP1 can self-assemble as well, their stability and size homogeneity are greatly improved when VP2 is co-expressed [32]. Furthermore, it may be that the stabilizing role of VP2 is essential for the diarrheagenic potential of VP1 [33] [34]. This might explain why we did not observe a marked effect from VP1-only VLPs on the intestinal motility. It would therefore be of further interest to assess the effect of VP2 as an individual protein on the induction of intestinal motility. However, individual expression or isolation of VP2 to sufficient yields is technically challenging.

Conversely, there is accumulating evidence favouring VP1 as the key determinant of diarrheagenic potential of norovirus strains. More specifically, in MNV, environmental triggers such as pH and the presence of bile acids evoke contraction of the VP1 P domain, resulting in increased interaction with the CD300lf receptor. A significant difference in reaction to these environmental cues was observed between MNV strains, resulting in different primary target cells and determining the diarrheagenic potential of the strains [6]. A similar flexible conformation of VP1 has been observed in HuNoV GII.4 in response to the presence of a metal ion [35]. Moreover, HuNoV VP1 has been shown to promote aquaporin (AQP) expression, which is a family of cell membrane pores with a central role in the regulation of water balance. Abnormal expression of AQP is known to be a direct cause of diarrhea [36]. In contrast to promoting AQP expression, HuNoV-triggered PKD2 phosphorylation can also suppress AQP3 expression, contributing to a disturbed expression pattern. This was recently described as one of the potential underlying mechanisms of HuNoV-induced diarrhea [37]. The enterotoxigenic potential of viral capsid proteins was already shown for astrovirus, another gastroenteritis-causing virus. Oral administration of purified astrovirus VLPs dose-and time dependently induced diarrhea in turkey poults [11].

Remarkably, we found that ip-injection of GII.6 VLPs containing VP1 and VP2 resulted in a comparable increase in gut motility as observed for the GII.4 VP1/VP2 VLPs, whereas yolk-injection of GII.6-containing stool did not elicit this effect. Infection of zebrafish larvae, after yolk-injection with HuNoV GII.6, results in lower viral titers than infection with HuNoV GII.4 [13]. This clarifies the observed difference in gut motility after infection with the two genotypes. On the contrary, when VP1/VP2 VLPs of the GII.4 and GII.6 genotypes were infected intraperitoneally at the same (high) concentration, the discrepancy in replicative efficiency is omitted, and both genotypes elicit a similar response.

Finally, since the viral capsid was able to induce increased motility, we checked whether this was already apparent shortly after ip injection of HuNoV positive stool samples, akin done with the VLPs. We observed a strong response 4h after HuNoV GII.4 ip injection, but little to no effect after ip injection of HuNoV GII.6 or GI.7 viruses. Hence, complete GII.4 virions are able to increase intestinal contractions at the used inoculum, while other HuNoV do not seem to do this (at least at the tested levels).

HuNoV GII.4 variants are the most common cause for HuNoV infections. Several variations in their virion morphology and antigenicity may explain their advantage over other HuNoV genotypes. For example, HuNoV GII.4 VLPs contain several GII.4-specific blockade epitopes of which some are conformation-dependent [38]. Epitope accessibility and particle formation are most likely dependent on several environmental factors, besides the primary genetic sequence, such as the type of host cell and temperature [39]. On top of that, GII.4 lineage viruses are known to have higher mutation rates in their genomes, giving rise to broader receptor binding capabilities and nucleotide incorporation rates, increasing viral fitness and helping GII.4 viruses escape the human immune system [40–42].This increased virion and capsid protein plasticity might explain the global dominance of HuNoV GII.4 and the reason we did not observe similar results with other genotypes after yolk injection, particularly given this is linked to a higher replicative efficiency.

A limitation of the present study is that we cannot entirely exclude the hypothesis that a non-viral component from the HuNoV-positive stool samples could elicit part of the observed gut motility. Given that an adequate viral stock has not been produced so far, we had to start infection using a positive stool sample. To mitigate the risk of a non-HuNoV specific effect in motility, we included negative controls (PBS and negative stool samples). While the used negative stool samples might not contain all possible non-viral factors present in the positive samples, their resulting negative data align with the theory of HuNoV-induced gut motility. An extra suitable control would be to block viral replication by means of a specific antiviral, i.e. 2’-C-methylcytidine (2CMC). However, we found that treatment with 2CMC alone also had an effect on intestinal motility (**S6 Fig**). This can be explained by the previously reported gastrointestinal side effects of 2CMC, among which vomiting at higher doses, which halted its further clinical development for the treatment of patients chronically infected with the hepatitis C virus [43]. In the absence of another equally efficient antiviral, the use of HuNoV-neutralizing antibodies would be a suitable alternative. On the contrary, UV-inactivation of the HuNoV-positive stool samples would not eliminate all viral capsids, for which we here present a dose-dependent effect on motility, and even would generate excessive reactive oxygen species [44], which could also impact measurements.

Overall, increased intestinal motility is not necessarily the main or only manifestation of HuNoV-induced disease, but is likely a single factor contributing to a broader, multifaceted effect of HuNoV on the gastrointestinal tract, leading to the classic clinical symptoms seen in humans [45]. We here show that HuNoV infection in zebrafish larvae affects their intestinal motility leading to increased, uncoordinated contractions in the intestinal bulb. These uncoordinated contractions result in a prolonged transit time of food particles. Moreover, we suggest that the viral capsid is responsible for inducing these changes in intestinal motility and thus may work as an enterotoxin. The minor protein VP2 plays a vital role in this process, either in a direct or indirect way. Further use of this model will advance the knowledge on HuNoV pathogenesis and the underlying mechanisms responsible for inducing the commonly seen clinical symptoms, vomiting and diarrhea, in humans.

## Material and methods

### Ethics statement

All zebrafish experiments were approved and performed according to the rules and regulations of the Ethical Committee of KU Leuven (P142/2021) in compliance with the regulations of the European Union (EU) concerning the welfare of laboratory animals as declared in Directive 2010/63/EU. Stool samples positive for HuNoV were obtained from the University Hospital of Leuven (UZ Leuven, Belgium) according to the rules and regulations of the Ethical Committee of KU Leuven (G-2021-4376) and the UZ Leuven (s63536).

### Zebrafish husbandry and maintenance

Adult wild-type AB zebrafish were housed in the Aquatic Facility of the KU Leuven at a temperature of 28°C with a 14h light/10h dark cycle. Fertilized eggs were obtained from adult zebrafish in mating tanks, and collected fertilized eggs were maintained in petri dishes (140 × 20.6 mm) with Danieau’s solution [1.5 mM HEPES buffer, 0.12 mM MgSO_4_, 0.18 mM Ca(NO_3_)_2_, 0.21 mM KCl, 17.4 mM NaCl, and 0.6 μM methylene blue] in an incubator, set at 28°C with a 14h light/10h dark cycle. At 72 hpf, all larvae were moved to another incubator set at 32°C to keep development constant in both HuNoV-infected and non-infected larvae.

### HuNoV VLP and protein expression

Proteins were expressed in *Spodoptera frugiperda* sf9 insect cells using the baculovirus expression system (ThermoFisher scientific). The genes were ordered from GeneArt services (ThermoFisher scientific) cloned in the pFastBac1 vector (**S1 Table**). To facilitate the purification process, the NS proteins included a GP67 exportation signal to the media and a FLAG Tag, both in amino terminal. The baculovirus expression vectors pFastBac1 were transformed into DH10Bac competent cells and the recombinant baculovirus were produced following the instructions manual (Bac-to-Bac, ThermoFisher scientific). The recombinant baculoviruses were utilized to produce the NS proteins or the VLPs. Briefly, Sf9 cells were grown in SF900SFMII media supplemented with antibiotics and 1% Pluronic (SIGMA) in suspension in spin flasks at 120 rpm and 27°C. The cells were subcultured to a density of 300,000 cells/ml and infected at an M.O.I. of two when they reached a density of 1,500,000 cells/ml. The cell supernatants were removed after 72 hours and the proteins were purified from the media. The NS proteins were purified using the M2 anti-flag affinity gel (SIGMA) according to the manufacturer’s protocol. Prior to the affinity purification, the media containing the recombinant proteins were ultra-centrifuged at 100,000 g using the R25ST rotor in a Himac CR-30N (Hitachi) to remove the recombinant baculoviruses and cellular debris. The recombinant proteins were eluted from the column with 0.1 mg/ml of soluble FLAG peptide (DYKDDDDK) that was subsequently removed from the protein preparation by successive ultra-filtration in 10KDa Amicon Ultra devices (Millipore). In a typical purification, 1 mg of recombinant NS protein was obtained from each liter of infected cells with the exception of NS3 and NS5 that unfortunately could not be expressed in the insect cells expression system. The production of VLPs was achieved as described above with some differences. The VLPs were precipitated from the first step of ultracentrifuged media with the addition of 15% PEG 8000 and 0.3 M NaCl to the supernatant. The mixture was incubated under agitation overnight at 4°C and then centrifuged at 10,000 g using the R16A rotor in a Himac CR-30N (Hitachi). The pellet was resuspended in PBS containing 1% Triton X100 (Merck), DNAse I (1 U/mL) and 1mM PMSF and incubated at room temperature for 30 minutes. Finally, the VLPs were ultra-centrifuged at 197,000 g using the SW41 rotor in a Beckman L8-M ultracentrifuge overnight at 4°C and the pellet containing the ultrapure VLPs was resuspended in PBS. The production of VLPs containing VP1 and VP2 was promoted with the infection of the SF9 cells with a mixture of baculovirus of VP1 and VP2.

### HuNoV VLP transmission electronic microscope imaging

15 μl of 0.1 mg/ml VLPs suspension in PBS was collected and incubated on collodion-carbon-coated copper grids. These grids were previously ionized by ion discharge on the Quorum Q150T ES system (Quorum Technologies, East Grinsted, UK). After 5 min of incubation on the grid, two successive washes were performed over 50 μl of MilliQ water. Finally, the sample was stained for 1 min with 5 μl of 2% (w/v) uranyl acetate. A FEI Tecnai 12 electron microscope equipped with a LaB6 filament, operated at 120 kV and equipped with a FEI Ceta CCD camera was used to observe the sample. The images were taken at a nominal magnification of 21,000 X.

### Coomassie blue staining

The recombinant proteins (4 μg/well) that represented each individual NS or VLP were analyzed by sodium dodecyl sulfate 12% polyacrylamide gel electrophoresis (SDS-PAGE) under reducing conditions and stained with coomassie blue.

### HuNoV and protein injection of zebrafish larvae

Yolk microinjections were performed as described previously [46]. Briefly, 3-dpf zebrafish larvae were anesthetized by immersion in 0.4 mg/mL tris-buffered tricaine in Danieau’s solution (Sigma-Aldrich, St. Louis, MO). The zebrafish larvae were then positioned in a 2% (wt/vol) agarose mold and infected with HuNoV via a 3 nL microinjection in the yolk. Infected larvae were recovered in Danieau’s solution in a 6-well plate with up to 20 larvae/well and kept at 32°C in a 14h-light/10h dark cycle for up to 3 dpi.

Intraperitoneal injection of HuNoV PBS samples, viral proteins and VLPs were performed on 5-dpf zebrafish with developed swim bladders. Briefly, larvae were anesthetized in tris-buffered tricaine and positioned laterally on a petri dish lid. Then, 2 nL of the appropriate solution was injected in the peritoneal cavity by inserting the needle posteriorly and ventrolateral of the swim bladder. Larvae were afterwards recovered in 6-well plates containing clean Danieau’s. Proteins and VLPs were injected at the following concentrations:

**Table:** 

**Protein**	**Concentration (mg/mL)**
**NS1/2**	3.1
**NS4**	1.78
**NS6**	2.6
**NS7**	7.45
**GII.4 VLP VP1**	2.5
**GII.4 VLP VP1 + VP2**	2.5
**GII.6 VLP VP1 + VP2**	2.5

### Intestinal motility imaging and analysis

Five-day old zebrafish larvae were screened for an empty intestinal tract to avoid triggering food-induced contractions. Larvae were subsequently anesthetized in 0.4 mg/ml tris-buffered tricaine and positioned laterally in 2% low-melting agarose in an 8-well μ-slide (Ibidi, Gräfelfing, Germany). Next, larvae were imaged for 5 minutes (1 image/s) using a Leica DMi8 inverted fluorescence microscope (Leica Microsystems, Wetzlar, Germany) and processed with the associated Leica Application Suite X (LAS X) software. Images were subsequently exported as a video (12 frames/s) for Danioscope analysis, or as individual frames for Igor Pro analysis. In Danioscope, videos were analysed using the flow analysis tool in hand drawn regions of interest in the intestinal bulb and posterior intestine (**S1 Fig**). Raw data was subsequently exported and plotted to determine period between peaks. For Igor Pro, analysis was performed using a custom script kindly provided by professor Julia Dallman [47]. Igor Pro values were used to plot data.

### Fluorescent food transit assay

Five-dpf larvae were fed a mixture 1 μm melamine resin rhodamine B-marked beads (Sigma-Aldrich, Missouri, USA), 100 μm larval food (SDS100, Special Diets Services, Augy, France) and sterilized water for 4h [48]. Pulverized dried food mixture was added to a 6-well plate containing larvae in approximately 4 mL Danieau’s. After 4h, larvae were put in clean water and individually screened for full intestinal bulbs containing the fluorescent mixture. Selected larvae were imaged which from then on was considered T_0_. Larvae were consequently imaged 1, 2, 3 and 4h after feeding. In between images, larvae were recovering in regular Danieau’s or Danieau’s containing compound (20 μM atropine, 200 μM serotonin, 500 μM emetine). Alternatively, larvae were only imaged again at 24h after feeding. Images were analyzed using FIJI-ImageJ where a region of interest encompassing the entire intestinal tract was determined and used to quantify fluorescent signal. Integrated density signal was used to calculate the evolution of the fluorescent signal over time, normalized to the T_0_.

### Statistical analysis

Statistical analysis was performed using Graphpad 10. Mann-Whitney test were used unless otherwise stated in the figure legends.

## Supporting information

S1 Fig **A)** An agar-embedded 5-dpf zebrafish analysed in Daniscope with the area of analysis defined by the two yellow boxes. Contraction frequency was analyzed in the intestinal bulb (IB) and posterior intestine (PI). **B)** Representative data output from the IB region of analysis. Graphs are automatically plotted by the software based on the change in pixel intensity (y-axis) over time of the video (x-axis).
(TIF)

S2 Fig Viral RNA copies of HuNoV GII.4 and HuNoV GII.6 at 2 days post infection. (n = 3–10). Mean with SD are presented. Viral RNA copies were quantified by RT-qPCR.
(TIF)

S3 Fig Zebrafish larvae with fully filled intestinal bulb after 4h of feeding. (TIF)

S4 Fig VLPs Transmission electronic Microscope images. The images show VLPs composed of A) VP1+VP2 or B) VP1 proteins, captured in a FEI Tecnai 12 electron microscope with a nominal magnification of 21,000 X.
(TIF)

S5 Fig Coomassie blue-stained 12% SDS-PAGE showing the different NS proteins and VLPs used in the present study. The name of each one of the proteins is indicated as well as the molecular weight marker (Mw). The molecular weights (in kDa) of the marker are indicated at the right of the gel. The expected molecular weight of each of the proteins are as follows: NS1/2 = 37 kDa; NS4 = 21 kDa; NS6 = 20 kDa; NS7 = 57 KDa, VP1 = 59 kDa and VP2 = 28 kDa. The molecular weights of proteins are calculated exclusively based on the mass of the amino acids present in the protein, excluding any potential posttranslational modifications. Due to its lower proportion to VP1, the VP2 can only be detected in the VLPs using Western Blotting [49].
(TIF)

S6 Fig 2CMC induces increased gut contractions. **A)** 2CMC successfully inhibited HuNoV GII.4 replication in zebrafish larvae. **B)** 2CMC increases gut motility, both in PBS- and HuNoV GII.4-injected larvae. *p ≤ 0.1, ** p ≤ 0.01.
(TIF)

S1 Table Amino acid sequences of the proteins utilized in the present study. Non structural proteins (NS1/2 to NS7) were cloned with and N-terminal GP67 exportation signal (italic) and Flag-Tagged (bold) to facilitate its purification. During the expression process, the cellular machinery removes the GP67 signal, cleaving it between the two last alanines. Structural proteins VP1 and VP2 were expressed without any tag.
(TIF)
