# Supplementary figures and images for: Human norovirus disturbs intestinal motility and transit time through its capsid proteins
Source: PLoS Pathog. 2024 Nov 27;20(11):e1012710. doi: 10.1371/journal.ppat.1012710 (PMC11602112; doi:10.1371/journal.ppat.1012710)

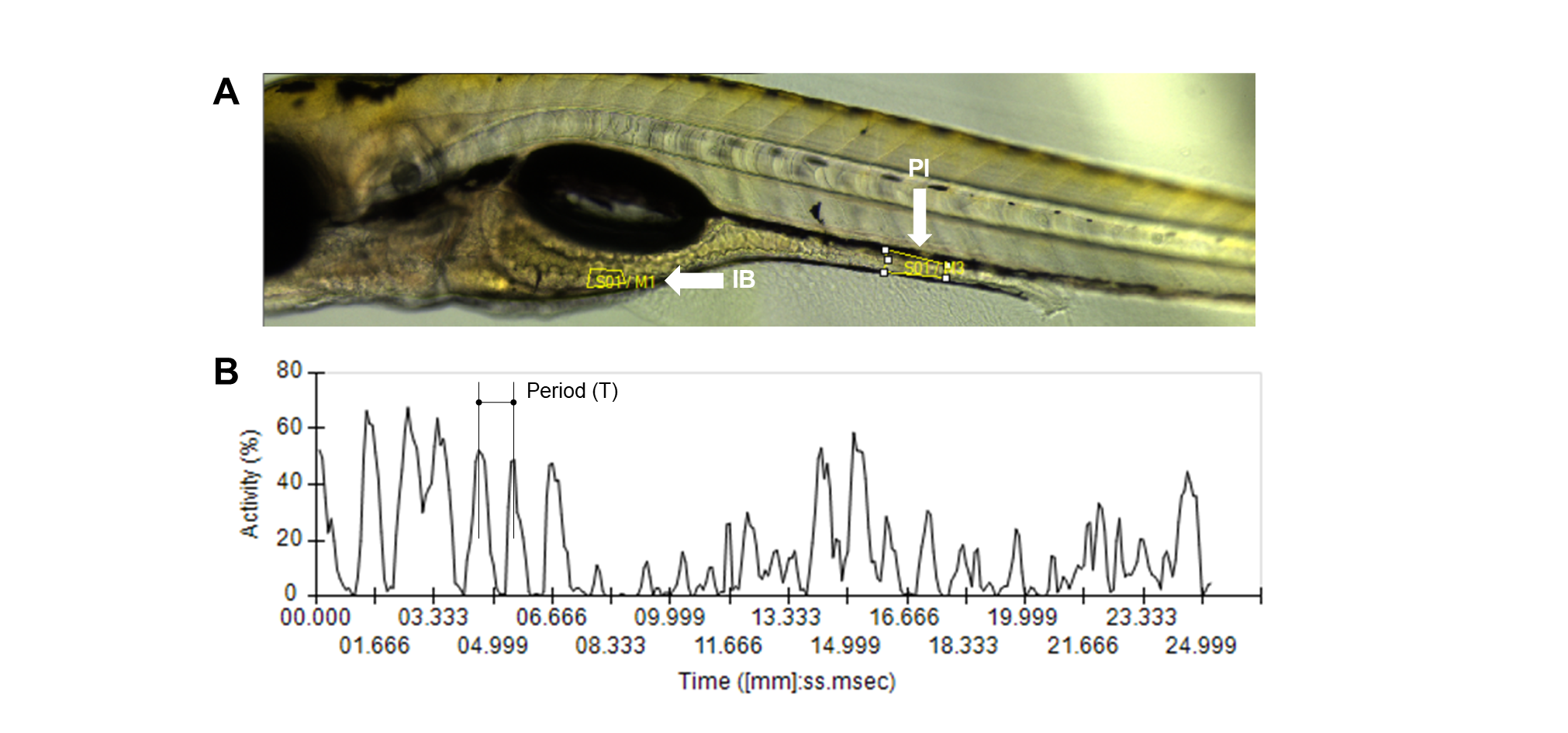

Supplement: S1 Fig — A) An agar-embedded 5-dpf zebrafish analysed in Daniscope with the area of analysis defined by the two yellow boxes. Contraction frequency was analyzed in the intestinal bulb (IB) and posterior intestine (PI). B) Representative data output from the IB region of analysis. Graphs are automatically plotted by the software based on the change in pixel intensity (y-axis) over time of the video (x-axis). (TIF) [file ppat.1012710.s001.tif]

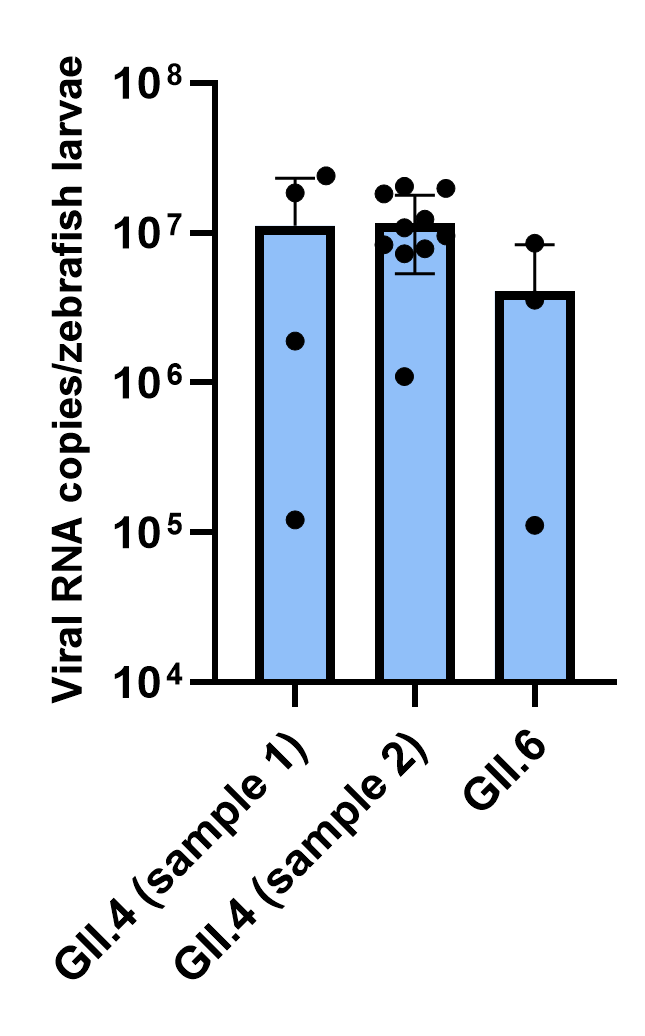

Supplement: S2 Fig — Viral RNA copies were quantified by RT-qPCR. (TIF) [file ppat.1012710.s002.tif]

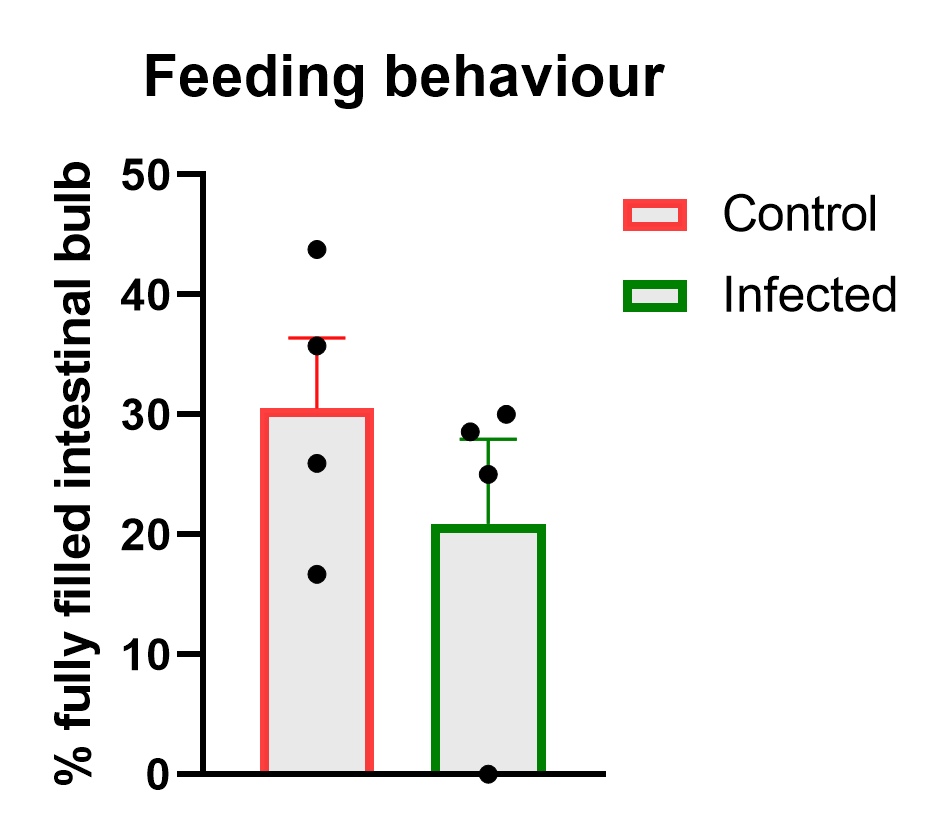

Supplement: S3 Fig — (TIF) [file ppat.1012710.s003.tif]

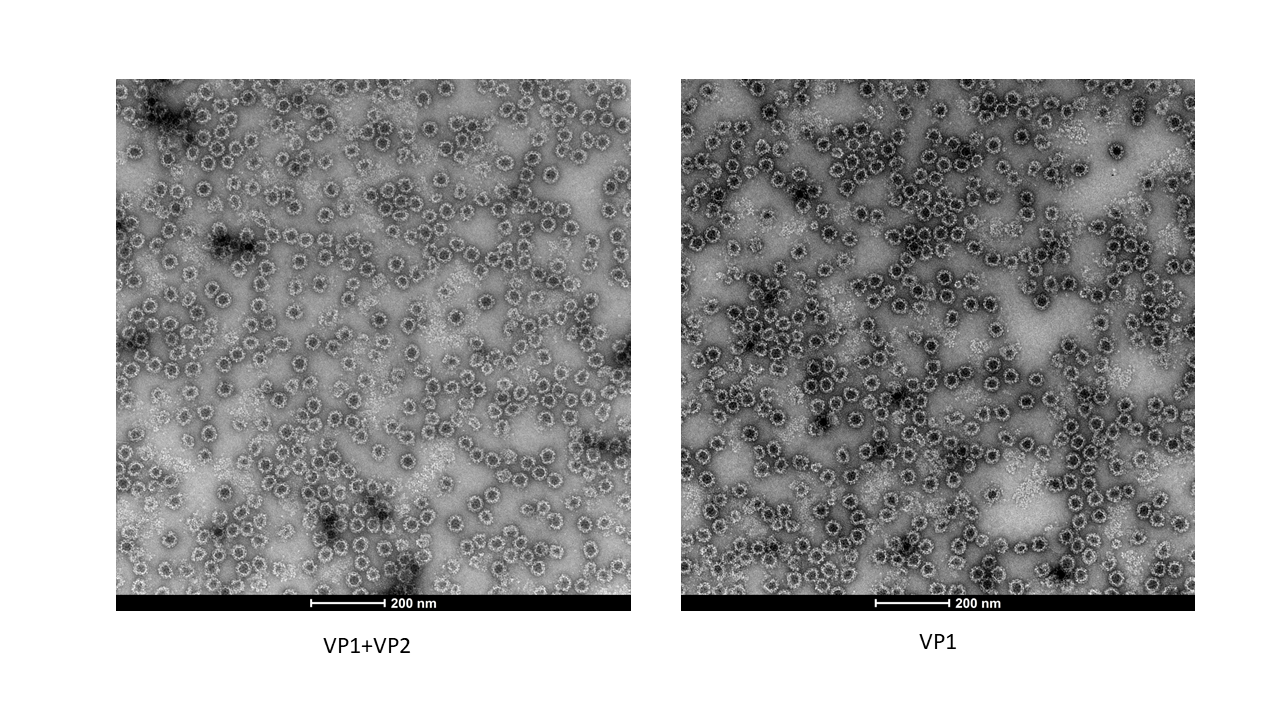

Supplement: S4 Fig — The images show VLPs composed of A) VP1+VP2 or B) VP1 proteins, captured in a FEI Tecnai 12 electron microscope with a nominal magnification of 21,000 X. (TIF) [file ppat.1012710.s004.tif]

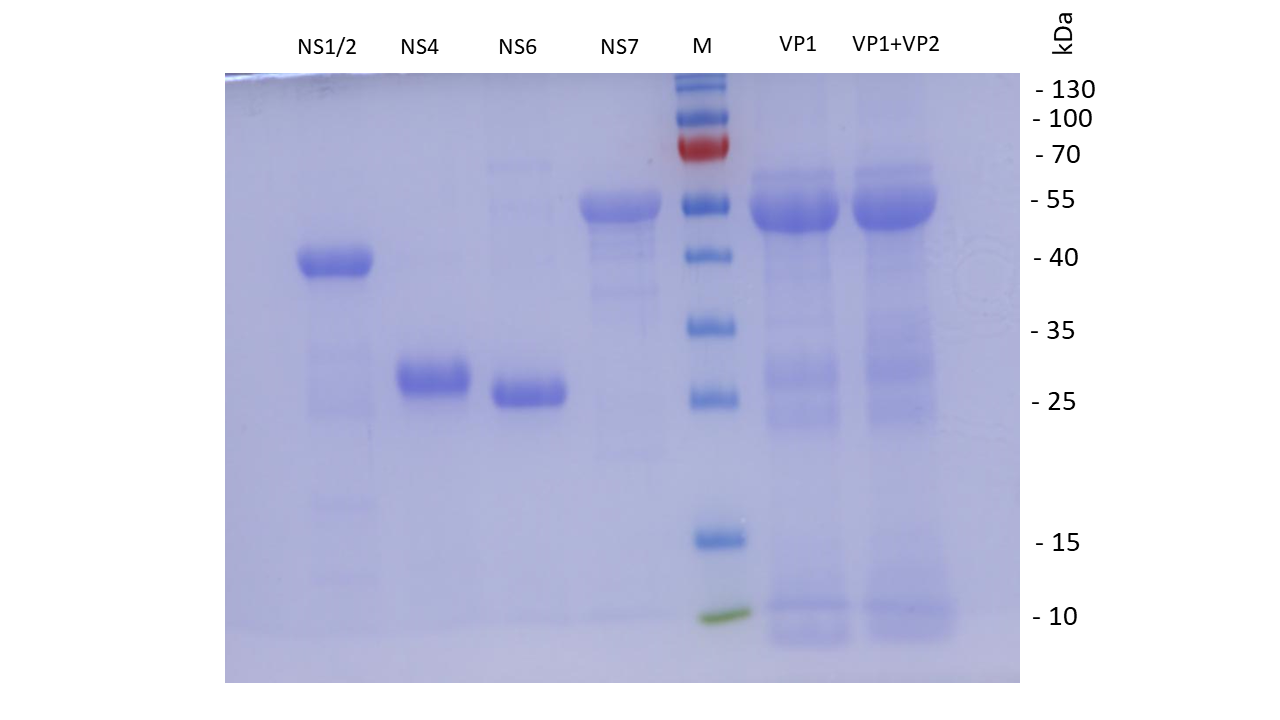

Supplement: S5 Fig — The name of each one of the proteins is indicated as well as the molecular weight marker (Mw). The molecular weights (in kDa) of the marker are indicated at the right of the gel. The expected molecular weight of each of the proteins are as follows: NS1/2 = 37 kDa; NS4 = 21 kDa; NS6 = 20 kDa; NS7 = 57 KDa, VP1 = 59 kDa and VP2 = 28 kDa. The molecular weights of proteins are calculated exclusively based on the mass of the amino acids present in the protein, excluding any potential posttranslational modifications. Due to its lower proportion to VP1, the VP2 can only be detected in the VLPs using Western Blotting [49]. (TIF) [file ppat.1012710.s005.tif]

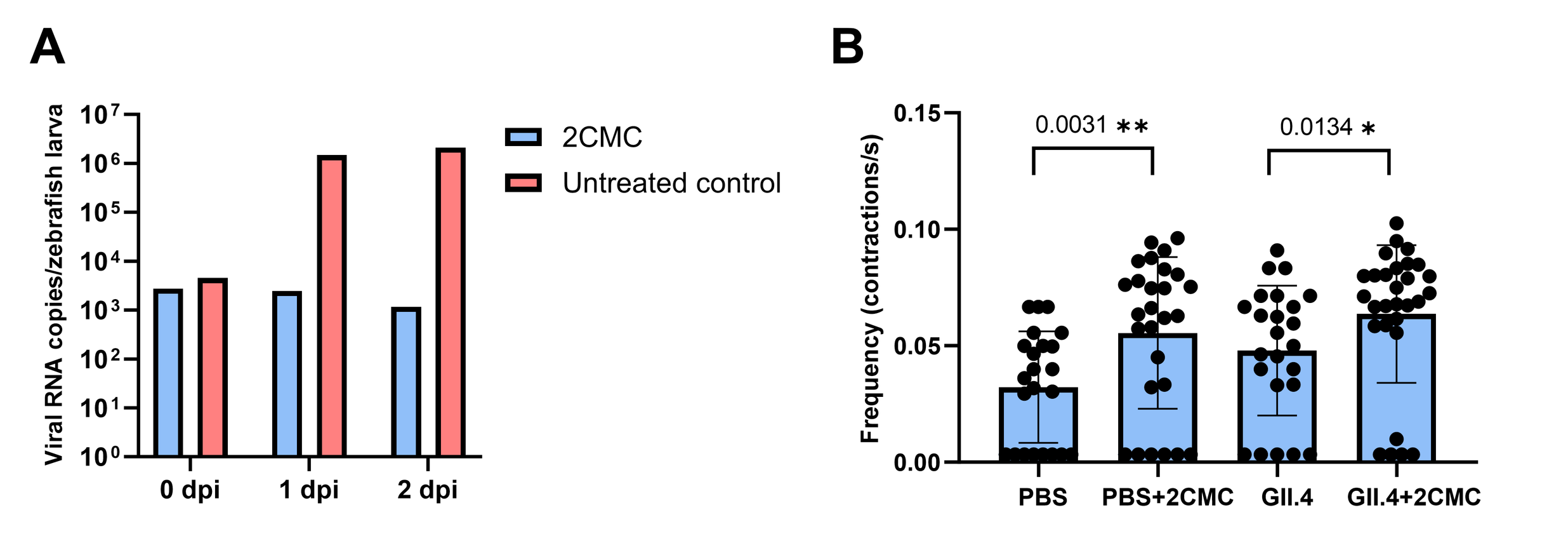

Supplement: S6 Fig — A) 2CMC successfully inhibited HuNoV GII.4 replication in zebrafish larvae. B) 2CMC increases gut motility, both in PBS- and HuNoV GII.4-injected larvae. *p ≤ 0.1, ** p ≤ 0.01. (TIF) [file ppat.1012710.s006.tif]

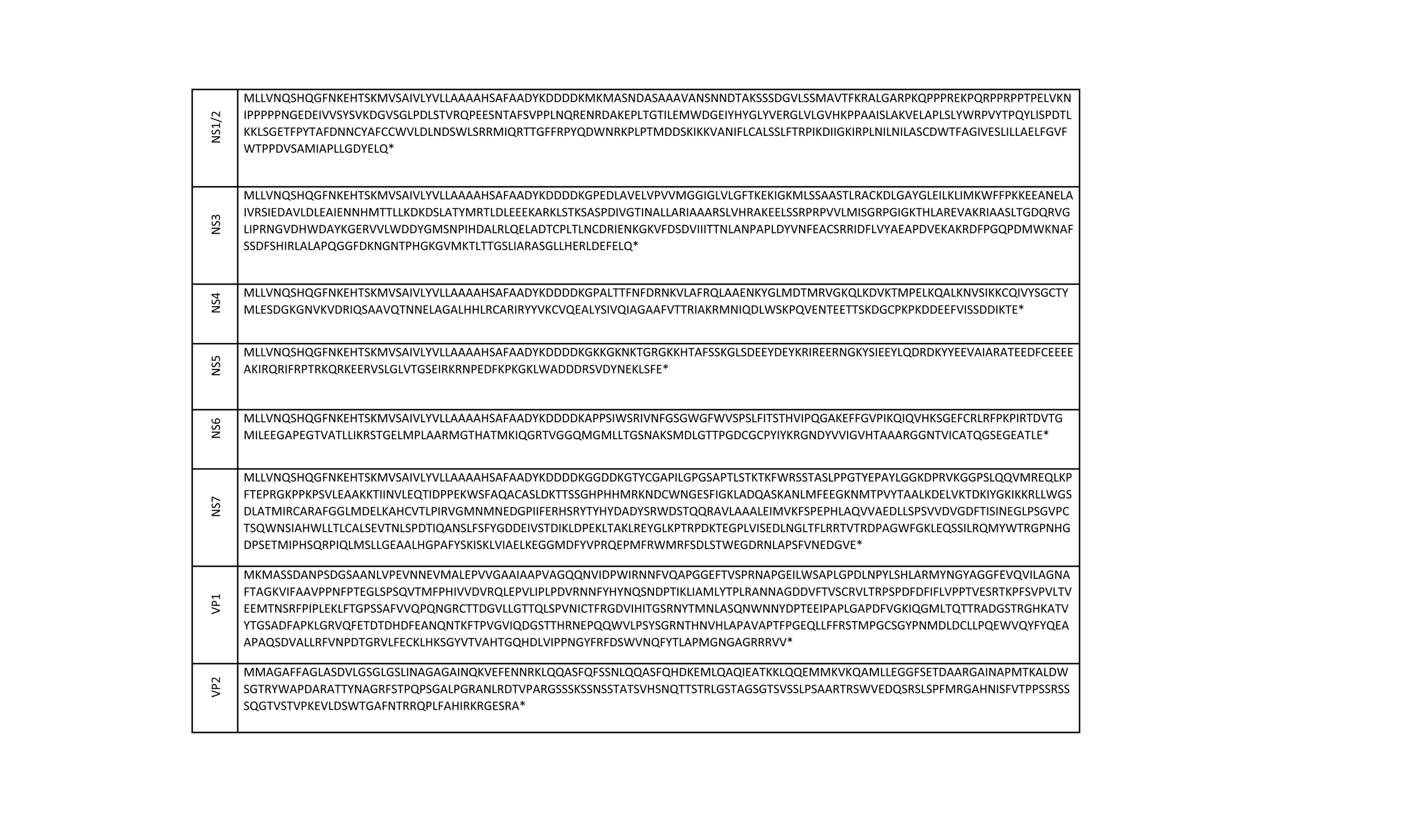

Supplement: S1 Table — Non structural proteins (NS1/2 to NS7) were cloned with and N-terminal GP67 exportation signal (italic) and Flag-Tagged (bold) to facilitate its purification. During the expression process, the cellular machinery removes the GP67 signal, cleaving it between the two last alanines. Structural proteins VP1 and VP2 were expressed without any tag. (TIF) [file ppat.1012710.s007.tif]
